# Leaf stoichiometric characteristics and responses of sea-buckthorn (*Hippophae* L.) involved in its niche-driven species distribution patterns across China

**DOI:** 10.3389/fpls.2025.1537643

**Published:** 2025-05-05

**Authors:** Hongmei Zhang, Xiaowei Li, Junlong Yang, Shuang Yu, Jun Yang, Wenqiang Wang

**Affiliations:** ^1^ College of Forestry and Pratacuture, Ningxia University, Yinchuan, China; ^2^ State Key Laboratory Breeding Base of Land Degradation and Ecological Restoration of Northwest China, Ningxia University, Yinchuan, China

**Keywords:** leaf stoichiometric characteristics, niche-driven species distribution, sea-buckthorn (*Hippophae L*.), Chinese sea-buckthorn (*Hippophae rhamnoides*), coastal sea-buckthorn (*Hippophae neurocarpa*), tibetan sea-buckthorn (*Hippophae tibetana*)

## Abstract

**Introduction:**

The stoichiometric characteristics of leaves can indicate the nutrient limitations and ecological strategies of closely related plants. Sea-buckthorn is a unique species in China with economic and ecological value, yet nutrient limitations and environmental adaptation strategies in its main distribution areas remain unclear.

**Methods:**

Here, This study aims to explore the adaptive mechanism in different geographical environments and its impact on species substitution distribution patterns by analyzing the leaf stoichiometric characteristics of three species of sea-buckthorn (*Hippophae rhamnoides, Hippophae tibetana and Hippophae neurocarpa*).

**Results:**

Our results reveal that the overall nitrogen (N) and phosphorus (P) concentrations of Sea-buckthorn leaves of are respectively 32.19 ± 4.96mg·g-1, and 2.03 ± mg·g-1 with an N/P ratio of 16.68 ± 4.01. Leaf N concentration significantly increased, with increasing longitude, and latitude. Leaf P concentration significantly increased, with the change of longitude. Among the three sea-buckthorn species, the N concentration of Chinese sea-buckthorn and Coastal sea-buckthorn exhibited increase significantly with latitude, whereas the N concentration of Tibetan sea-buckthorn showed significant variation with longitude. Through standardised major axis (SMA) regression, an allometric relationship was observed between the N and P concentrations across sea-buckthorn species. Notably, the N and P concentrations of Tibetan sea-buckthorn exhibited significant allometric relationship. Potential evapotranspiration and altitude were identified as the primary factors influencing N concentration, while slope and humidity index were the main drivers affecting P concentration. Additionally, average annual radiation and precipitation were found to influence the N/P ratio. The leaf stoichiometry of sea-buckthorn species revealed distinct adaptation mechanisms to their respective habitats, leading to niche-driven species distribution patterns along environmental gradients. Tibetan sea-buckthorn served as a transitional zone between the other two species.

**Discussion:**

Therefore, this study can provide basic data for the stoichiometric characteristics of sea-buckthorn leaves and the distribution pattern of species substitution, with provides a new ecological perspective on the relationship between species distribution and plant stoichiometric characteristics.

## 1 Introduction

Plant stoichiometry is mainly concerned with how the internal of elemental composition of plants changes with the environment, which drives their physiological and ecological processes and thus pertinent to understanding vegetation composition, ecosystem function, and nutrient limitation (Zhang et al., 2018; Tian et al., 2018; Lu et al., 2023). The elemental concentration of N and P are commonly utilized assess the nutritional or growth status of plants (Tao et al., 2016). The N/P ratio, serving as a critical indicator for assessing nutrient limitation and growth rates, can elucidate the ecological strategies adopted by plants. The rational allocation of elemental concentration within plants facilitates the maintenance of their stable metabolic capacity (Tao et al., 2016; Zhang et al., 2018). The allometric relationship acts as a foundational theory for describing resource allocation, and the allometric indices of nitrogen (N) and phosphorus (P) indicate the rates of change in N and P concentration within plants (Tian et al., 2018). However, the variation in elemental characteristics among plants is closely linked to the adaptation strategies utilized by different species in response to environmental conditions (Wu et al., 2024). Some studies have suggested that niche differentiation plays a crucial role in facilitating the coexistence of relatively smaller species within ecological communities (Wu et al., 2012). Topography, energy, and water are pivotal environmental factors that substantially influence the structure and function of ecosystems. These factors affect species distribution, community composition, and ecological processes (Yu et al., 2017). Under diverse environmental conditions, competition for resources and ecological niche differentiation lead to the establishment of distinct distribution patterns among different species, a phenomenon referred to as alternative distribution (Sterck et al., 2014). Although the phenomenon of species substitution distribution is widely acknowledged and prevalent, its underlying mechanisms remain inadequately understood.

Most studies on plant stoichiometric characteristics have focused on regional stoichiometric patterns and their driving factors. For example, studying the stoichiometric characteristics of leaves of Chinese flora Han et al. (2005), found that plant growth was limited by P, and the leaf N and P concentrations increased with increasing latitude. Tian et al. (2024) demonstrated that environmental factors significantly influence the responses of leaf nitrogen (N), phosphorus (P), and their N/P ratios within species. Specifically, leaf N and P concentrations tend to increase in cooler climates and mid-latitude arid regions, resulting in a subsequent decrease in the N/P ratio. Furthermore, Vasseur et al. (2023) and Ouyang et al. (2024) reported that the allometric scaling indices for plant N and P are 3/2 and 3/4, respectively. Ye et al. (2002) found that the distribution of plant species in subtropical and tropical mountains followed a pronounced substitution rule along a habitat gradient. There are research findings indicating that conspecific plants can exhibit close similarities in their leaf morphological structures and physiological-ecological traits. Even when distributed across different environmental regions, they can partially substitute for one another in space (and time), ultimately forming a geographically substituted distribution pattern (Korall and Pryer, 2014). Yet those studies did not provide a complete and definitive conclusion on exactly which factors influence plant stoichiometry, which has since prompted the proposal of multiple hypotheses to explain the observed variation in nature. The plant physiology hypothesis holds that metabolic processes in plants are sensitive to temperature, and a greater N or P concentration can compensate for a diminished metabolic rate under low temperature or high elevation conditions (Chen et al., 2013). The *species composition hypothesis* posits that differences in the composition of plants species or life forms affect the biogeographical patterning of leaf stoichiometric characteristics. According to the *biogeochemical niche hypothesis*, plants require a specific elemental composition to maintain their growth and species differentially occupy positions and sizes in high-dimensional space formed by multiple element concentrations; that is, ecological niches differing in their stoichiometric characteristics are distinct (Peñuelas et al., 2019). Nevertheless, these hypotheses generally assume that phytostoichiometry is influenced by different species, climate, and environmental factors. Therefore, elucidating the mechanisms responsible for shifts in the leaf N, P, and N/P is crucial for exploring the plant-based ecosystem processes in fluctuating environments, species adaptation to geographic variation, and ecosystem balance dynamics.

China is the diversity epicenter of sea-buckthorn, a kind of cold-resistant and drought-resistant woody plant, rich in a variety of nutrients, with various ecological, nutritional, economic, health, and tourism-related values. It has significant meaning and advantages in protecting the ecological environment and promoting sustainable development. This shrub, better known as sea-buckthorn, not only contributes substantially to enhancing the ecological environment but also to developing food and medicinal products (Li et al., 2014). Sea-buckthorn are natively distributed in North China, Northwest China, and Southwest China, being found primarily in the ecotone between forest and grassland, with species responding markedly to precipitation and concentrated in areas with an average annual rainfall of 400–700 mm distinguished by a longitudinal zonal distribution (Zhang et al., 2010; Li et al., 2014). Current studies on sea-buckthorn have mainly focused on its root nodal endophytes, leaf carbon stable isotopes, and genome involved in flavonoid synthesis pathways (e.g., Li et al., 2014; Guo et al., 2025). However, research on the stoichiometric characteristics and substitution distribution of sea-buckthorn leaves across different spatial scales remains limited. In this study, sea-buckthorn populations located in the Loess Plateau and the eastern margin of the Qinghai-Tibet Plateau were selected as the research subject to elucidate the influence of environmental factors on plant stoichiometric traits, investigate nitrogen (N) and phosphorus (P) nutrient cycles and adaptation strategies, and establish the relationship between the stoichiometric characteristics of sea-buckthorn plants and their species substitution distribution patterns. Specifically, this study aims to address the following questions: (1) What is the adaptation mechanism of leaf stoichiometry in sea-buckthorn under varying environmental conditions? (2) What are the key factors influencing the stoichiometric characteristics of sea-buckthorn leaves? (3) Does the stoichiometric profile of sea-buckthorn leaves affect the alternative distribution pattern of sea-buckthorn species formation?

## 2 Materials and methods

### 2.1 Study area

**Figure 1:**
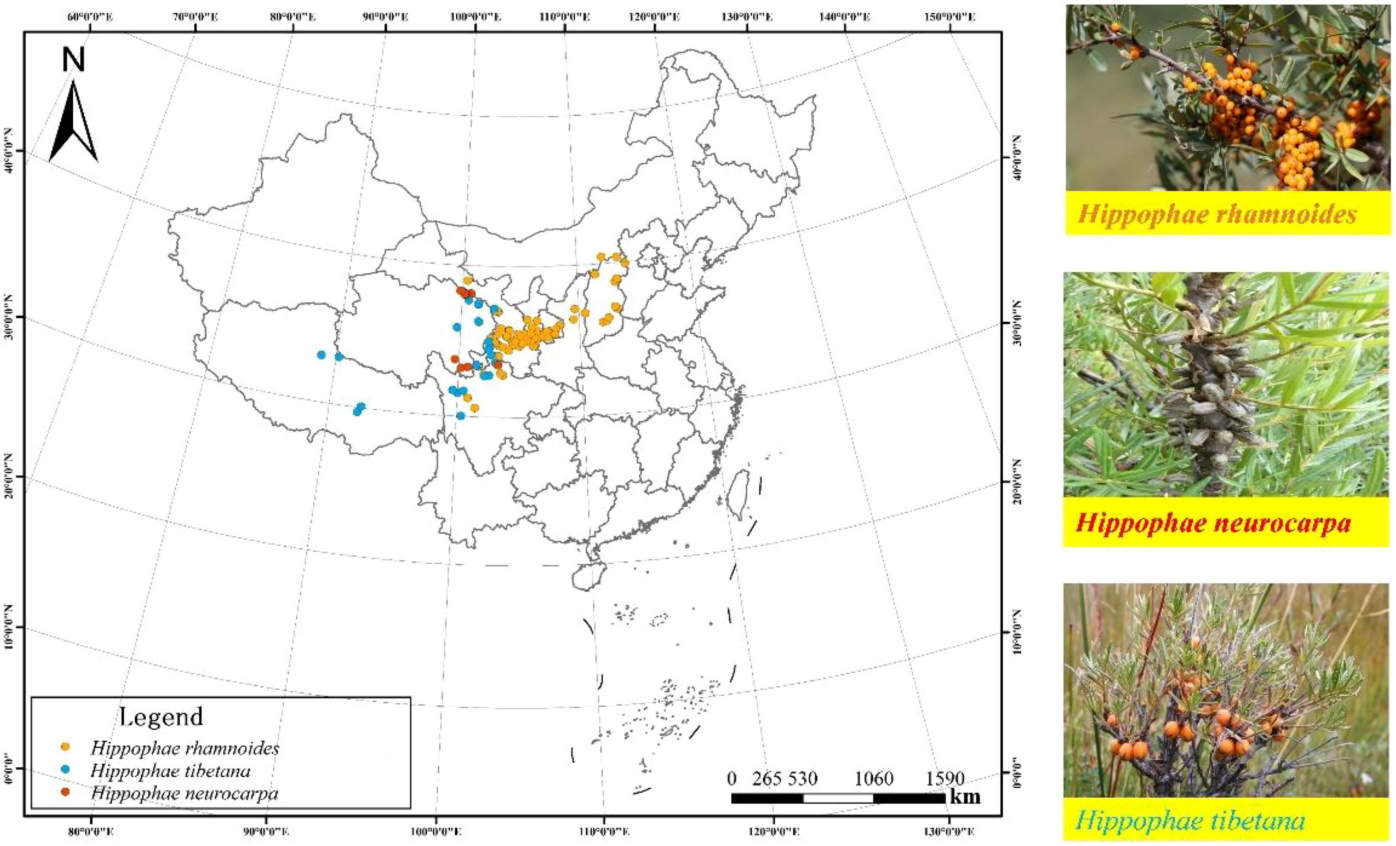
Map of the study area in China showing the locations of the 110 sampling sites for sea-buckthorn shrubs sea-buckthorn. The orange dots represent the Chinese sea-buckthorn (*Hippophae rhamnoides*), the blue dots represent the Tibetan sea-buckthorn (*Hippophae tibetana*), and the red dots represent the Coastal sea-buckthorn (*Hippophae rhamnoides*).

The study selected the natural populations of the Sea-buckthorn genus, which are primarily distributed across the Loess Plateau and the Qinghai-Tibet Plateau. The research focuses on three dominant species regionally distributed in China: Chinese sea-buckthorn, Tibetan sea-buckthorn, and Coastal sea-buckthorn. A total of 110 sampling sites were identified (**Figure 1**). Of these, 74 natural populations were for Chinese sea-buckthorn in the Loess Plateau and eastern margin of Qinghai-Tibet Plateau, covering the provinces of Hebei, Inner Mongolia, Shanxi, Shaanxi, Ningxia, Gansu and Qinghai (34°-40°N, 100°-114°E; 1024–3209 m a.s.l.). The 25 natural populations sampling sites of sea-buckthorn in Tibet were largely situated in the southeastern Qinghai-Tibet Plateau, spanning four provinces: Gansu, Qinghai, Tibet, and Sichuan (29°-38°N, 88°-103°E; 2869–4860 m a.s.l.). According to the native range of sea-buckthorn in the Loess Plateau and Qinghai-Tibet Plateau, 11 natural populations were selected as sampling sites. Average annual temperature (MAT) ranged from –5.87 to 4.53°C, and average annual precipitation (MAP) ranged from 222–766 mm (**Figure 1**).

### 2.2 Sampling and measurement

In each sampling site, a plot was set up (n = 110), in which sea-buckthorn leaves with good development, uniform growth, and lacking any disease symptoms were selected. For each species, 30 individual mature leaves were collected from different directions and mixed into a single sample, with three such leaf composite samples obtained per plot (330 leaf samples in total). Each sample was put into a labeled envelope and taken to the laboratory. There, all leaf samples were dried at 65°C to constant weight, crushed, and screened through 100-mesh to determine their respective N and P concentrations. The leaf N concentration was measured using the Kjeldahl method, while the leaf P concentration was measured with the molybdenum-antimony resistance colorimetric method (Jones, 2001).

### 2.3 Climatic variables

The climate data used in this study came from the WorldClim website (http://worldclim.org). This dataset provides global average monthly meteorological data, at 1km×1km resolution, for 30 recent years. Monthly data were obtained by inputting the coordinates of each sampling site (in **Figure 1**), for which the annual average data is then calculated, with respect to three sets of factors: energy, moisture, and terrain. The energy factors were the maximum temperature (MAXT), minimum temperature (MINT), average annual radiation (Ssrad), warmth index (WI). Among water factors were the potential evapotranspiration (PET), and humidity index (HI), average annual rainfall (MAP), and Vapor Pressure (Vapr) were considered here. Topographic factors included wind, altitude, aspect, and slope. ArcGIS 10.2 software was used to extract meteorological data for each sampled plot, according its latitude and longitude. Potential evapotranspiration (PET), warmth index (WI), and humidity index (HI) were calculated according to the methods of Zhang (1989) and Zhang et al. (2017).

### 2.4 Statistical analyses

In processing the data Excel 2019 software was used for preliminary data sorting, after which SPSS 25 software was used for formal data analyses. One-way analysis of variance (ANOVA) was used to compare the differences in N, P, and N/P response variables among the three sea-buckthorn species, for which Duncan’s test were used to carry out multiple *post-hoc* comparisons, with statistical significance set to α = 0.05. To investigate the spatial pattern and distribution relationship of plant leaf stoichiometry, linear regression analysis was employed. The R software package UpSetR was employed for the graphical analysis of the intersection of stratified visual sets (Conway et al., 2017). This approach facilitated the identification of key environmental factors influencing nitrogen (N) and phosphorus (P) concentrations in sea buckthorn, as well as the N/P ratio. The relationship between N and P can be described by a mathematical equation, y = βxα. The linear form is log (y) = log (β) + αlog (x), x and y determine whether the relationship is isometric (α = 1.0) or allometric (α > 1.0 or α < 1.0). Standardized major axis regression (SMA) was implemented using the ‘SMART’ package in R2.0 (Warton et al., 2010). Finally, redundancy analysis (RDA) analysis was performed using Canoco 5 software, to verify the species substitution distribution according to the spatially distributed stoichiometric characteristics of sea-buckthorn leaves along the environmental axes. Figures were drawn in the R v4.3.2 platform, Origin Pro 2021(Origin Lab, USA), and Canoco 5.

## 3 Results

### 3.1 Leaf stoichiometry characteristics in sea-buckthorn

**Figure 2:**
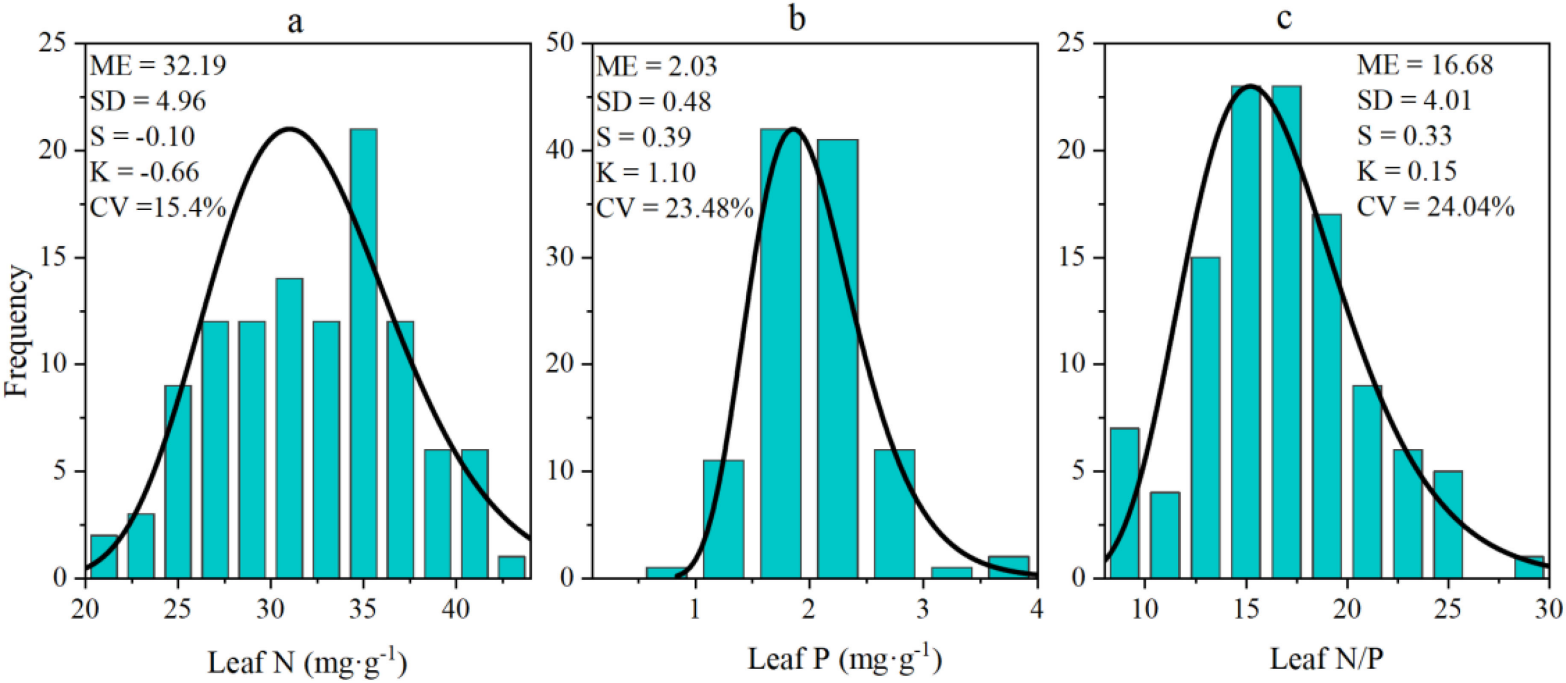
Histograms of N **(a)**, P **(b)**, and their N/Pratio **(c)** in leaves of sea-buckthorn shrubs (all three sea-buckthorn species pooled).

As **Figure 2** shows, for N, P, and N/P ratio of the sea-buckthorn were normally distributed and their skewness values were always less than 1 (Kolmogorov-Smirnov test, *P* > 0.05). The CV (coefficient of variation) values of N, P, and N/P respectively were 15.4%, 23.48%, and 24.04%. Among the three sea-buckthorn, the leaf N and P concentrations were higher in Chinese sea-buckthorn than Coastal sea-buckthorn, being lowest in Tibetan sea-buckthorn. However, the N/P ratio was highest in leaves of Tibetan sea-buckthorn (**Table 1**).

**Table 1:** Nitrogen and phosphorus concentrations and their ratio in sea-buckthorn leaves.

Species	Sample	N(mg·g^-1^)		P(mg·g^-1^)		N/P	
		Mean ± SE	CV(%)	Mean ± SE	CV(%)	Mean ± SE	CV(%)
*Hippophae rhamnoides*	n=74	33.60 ± 4.63a	13.69	2.16 ± 0.39a	17.89	16.16 ± 3.32a	20.41
*Hippophae tibetana*	n=25	28.41 ± 2.48b	8.57	1.66 ± 0.38b	22.35	17.85 ± 3.73a	20.49
*Hippophae neurocarpa*	n=11	31.28 ± 6.73ab	20.50	2.04 ± 0.76a	35.55	17.51 ± 7.42a	40.40
Significance test		*P* =0.0001		*P* = 0.0001		*P* = 0.14	
F		12.69***		12.14***		1.97	

Different lower-case letters within a column indicate significant differences between species. ***.P<0.001.

### 3.2 Spatial distribution pattern for leaf stoichiometric characteristics of sea-buckthorn

**Figure 3:**
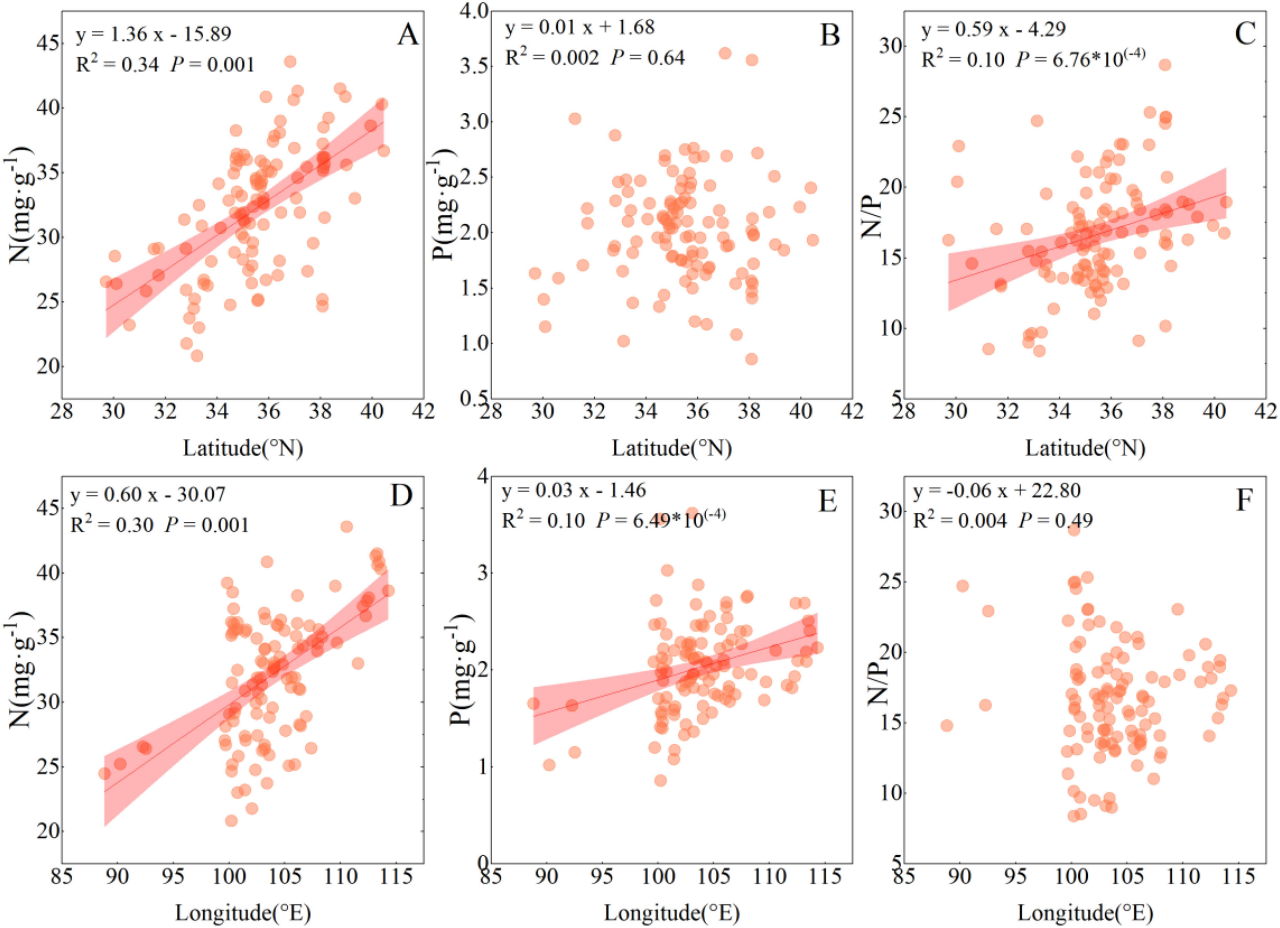
Spatial trends in the nitrogen and phosphorus concentrations and N/P ratio in leaves of sea-buckthorn genus. Shaded bands are the 95% confidence intervals for the fitted regression lines. Each symbol is a plot (n = 110). **(A–C)** represent the influences of latitude on the stoichiometric ratios of nitrogen (N), phosphorus (P), and the N/P ratio in the genus Sea-buckthorn, respectively. Similarly, **(D–F)** indicate the effects of longitude on the stoichiometric coefficients of nitrogen (N), phosphorus (P), and the N/P ratio in sea- buckthorn.

The leaf N concentration of sea-buckthorn was positively correlated with longitude, and latitude (*P* > 0.05). Their leaf P concentration was positively correlated with longitude (*P* < 0.001), while not significantly correlated with latitude (*P* > 0.05). The N/P ratio was significantly positively correlated with latitude (*P* < 0.001), albeit not significantly correlated with longitude (*P* > 0.05) (**Figure 3**).

**Figure 4:**
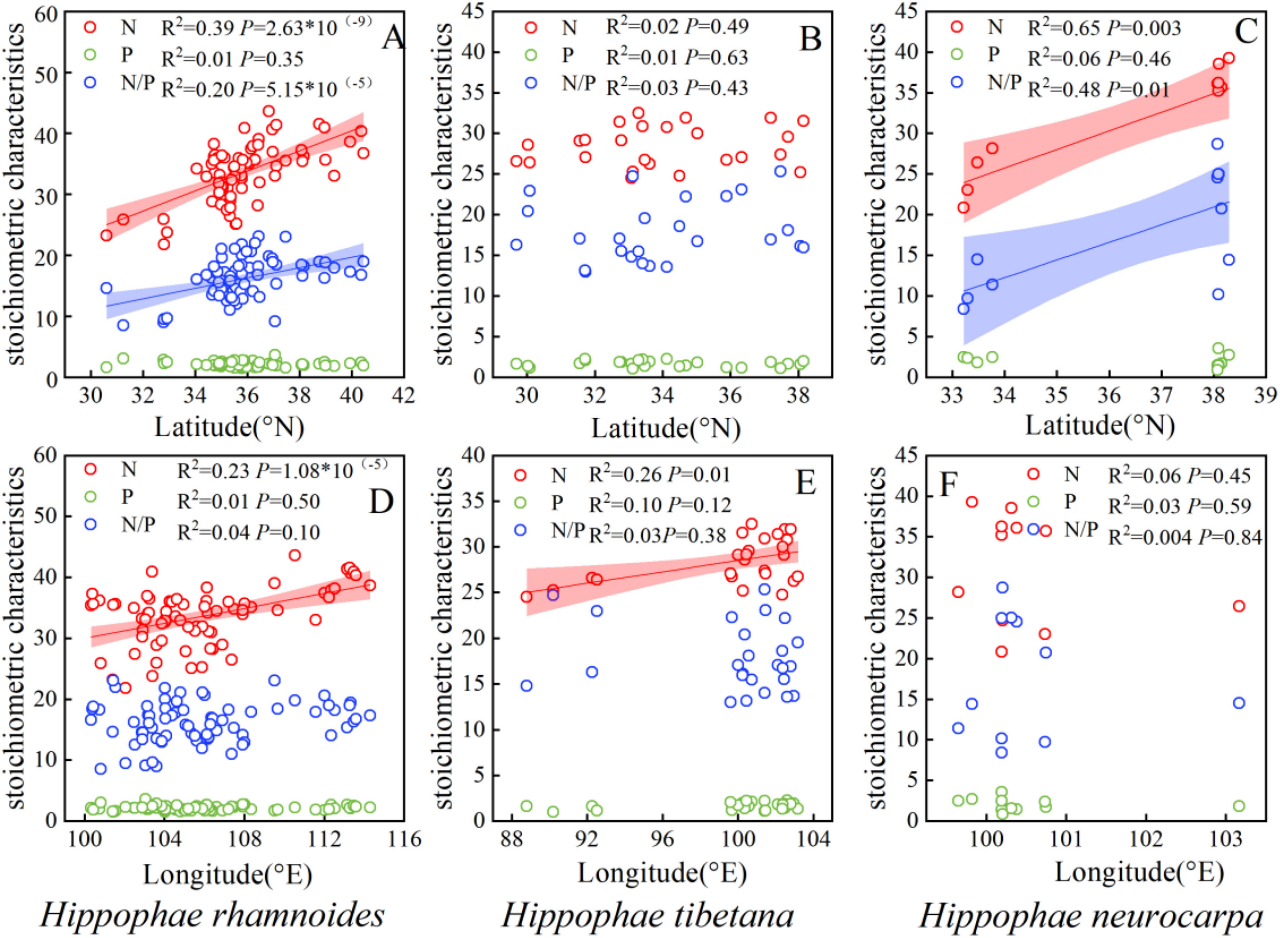
Spatial trends in the nitrogen and phosphorus concentrations and N/P ratio in leaves of different sea-buckthorn. Shaded bands are the 95% confidence intervals for the fitted regression lines. **(A, D)** illustrate the influence of latitude and longitude on the stoichiometry of sea-buckthorn leaves in China Chinese sea-buckthorn, **(B, E)** depict the influence of latitude and longitude on the stoichiometry of Tibetan sea-buckthorn, while **(C, F)** demonstrate the influence of latitude and longitude on the stoichiometry of Coastal sea-buckthorn.

The N concentration, and N/P ratio of Chinese sea-buckthorn and Coastal sea-buckthorn exhibited significant increases with rising latitude (*P* < 0.01). However, no significant variation was observed in the N/P ratio of Tibetan sea-buckthorn alone (*P* > 0.05). Additionally, the N concentration of both Chinese sea-buckthorn and Tibetan sea-buckthorn showed significant positive correlations with longitude (*P* < 0.01), (**Figure 4**).

### 3.3 Allometric relationship between the N and P in sea-buckthorn leaves

**Figure 5:**
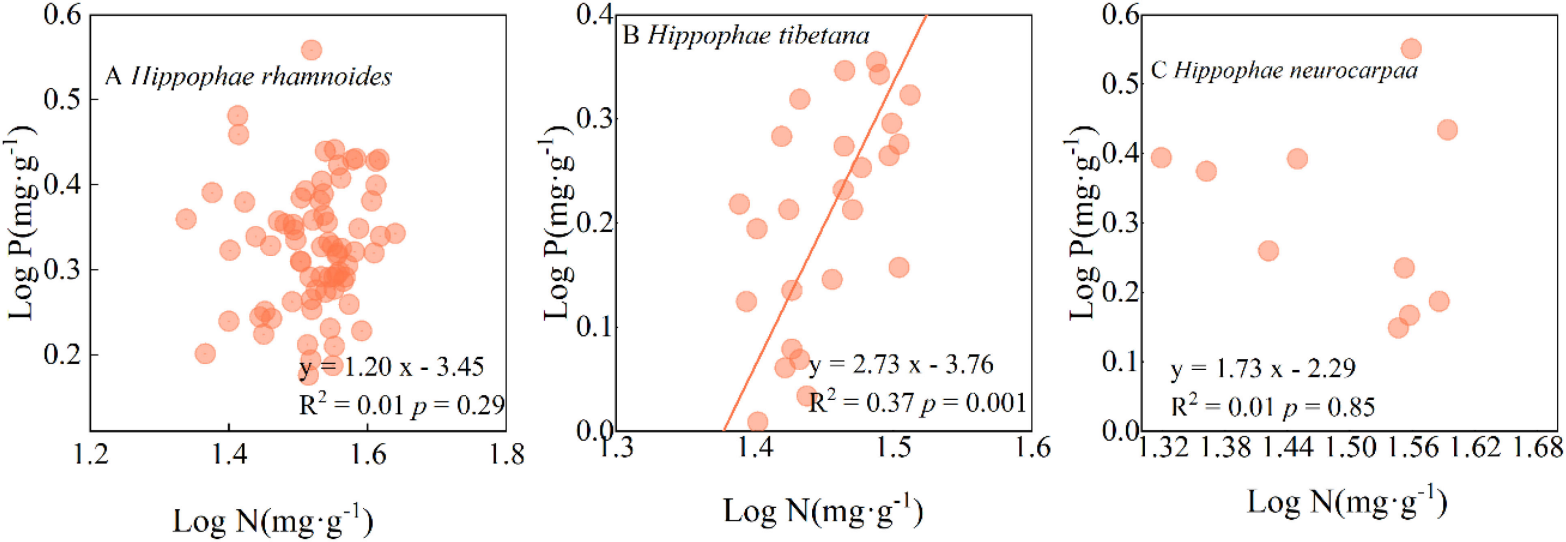
Relationship between the leaf N and P concentrations in different sea-buckthorn species.

The standardised major axis (SMA) regression demonstrated Tibetan sea-buckthorn the lack of a 1:1 correspondence between N and P, but there was nonetheless a significant allometric relationship (*P* < 0.01), with a slope of 2.73 (95%CI: 2.15, 3.46); Chinese sea-buckthorn species, and Coastal sea-buckthorn species between N and P no significant allometric relationship (*P* > 0.05) (**Figure 5**).

### 3.4 Factors influencing the N and P concentrations and N/P ratio of *Hippophae* leaves

**Figure 6:**
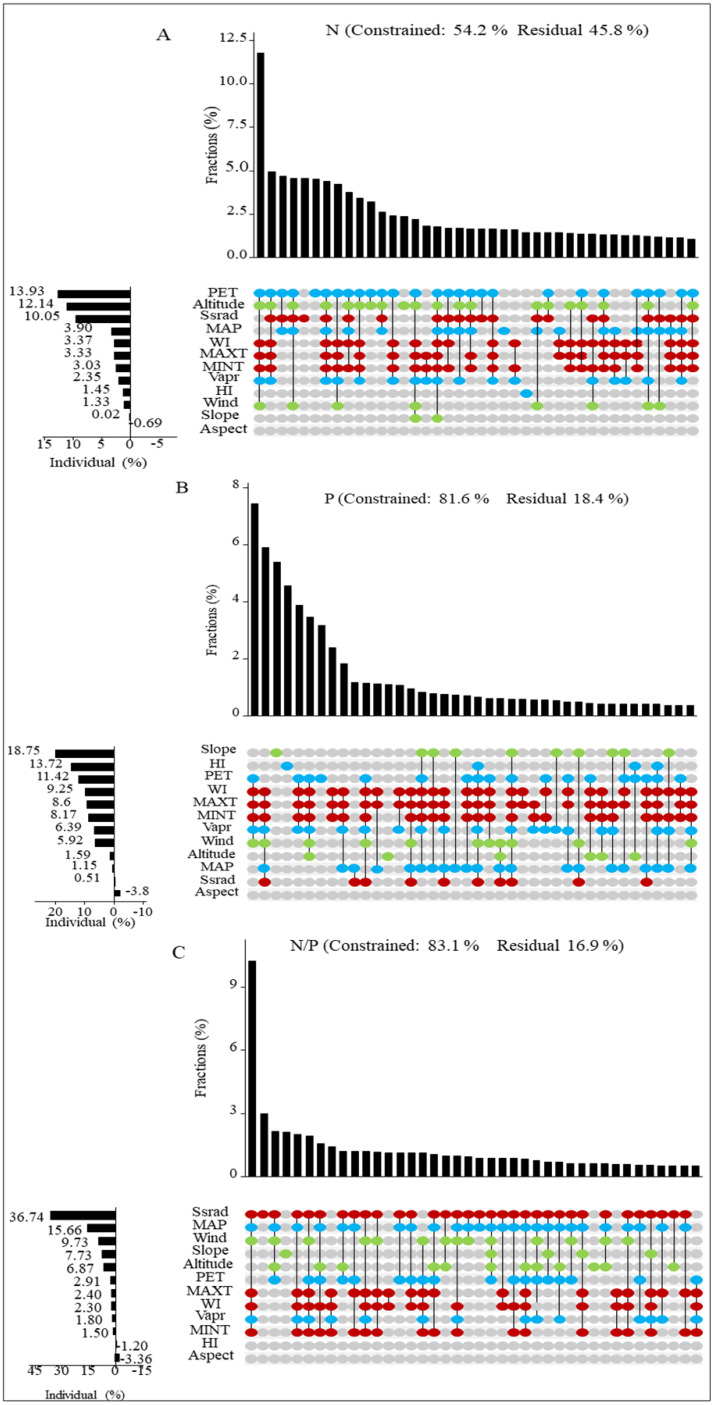
**(A–C)** respectively represent the interpretations of the stoichiometric characteristics of sea-buckthorn leaves (aggregated across three sea-buckthorn species) in relation to energy, water, and topography factors. (1) The red dots represents the energy factor, the blue dots represents the water factor, and the green dots represents the topographic factor. (2) Factors abbreviations are Maximum Temperature (MAXT), Minimum Temperature (MINT), Annual Mean Solar Radiation (Ssrad), Potential Evapotranspiration (PET), Warmth Index (WI), Humidity Index (HI), Mean Annual Precipitation (MAP), Vapor Pressure (Vapr), Altitude, Aspect, Slope.

To further investigate the influencing factors on the stoichiometry of sea-buckthorn leaves, we employed hierarchical segmentation to systematically prioritize and rank these factors. UpSet analysis revealed that PET and altitude were the predominant factors influencing the N concentration in sea-buckthorn, followed by Ssrad (**Figure 6A**). The primary factors affecting P concentration were slope and HI, with PET being secondary (**Figure 6B**). Additionally, the N/P ratio was influenced by Ssrad, MAP, and wind (**Figure 6C**). Overall, water-related factors were identified as the major drivers of N and P concentrations (21.63% and 32.68%, respectively), while energy-related factors predominantly affected the N/P ratio (42.94%).

### 3.5 Relationships between stoichiometric characteristics and geographical substitution distribution of sea-buckthorn leaves

**Figure 7:**
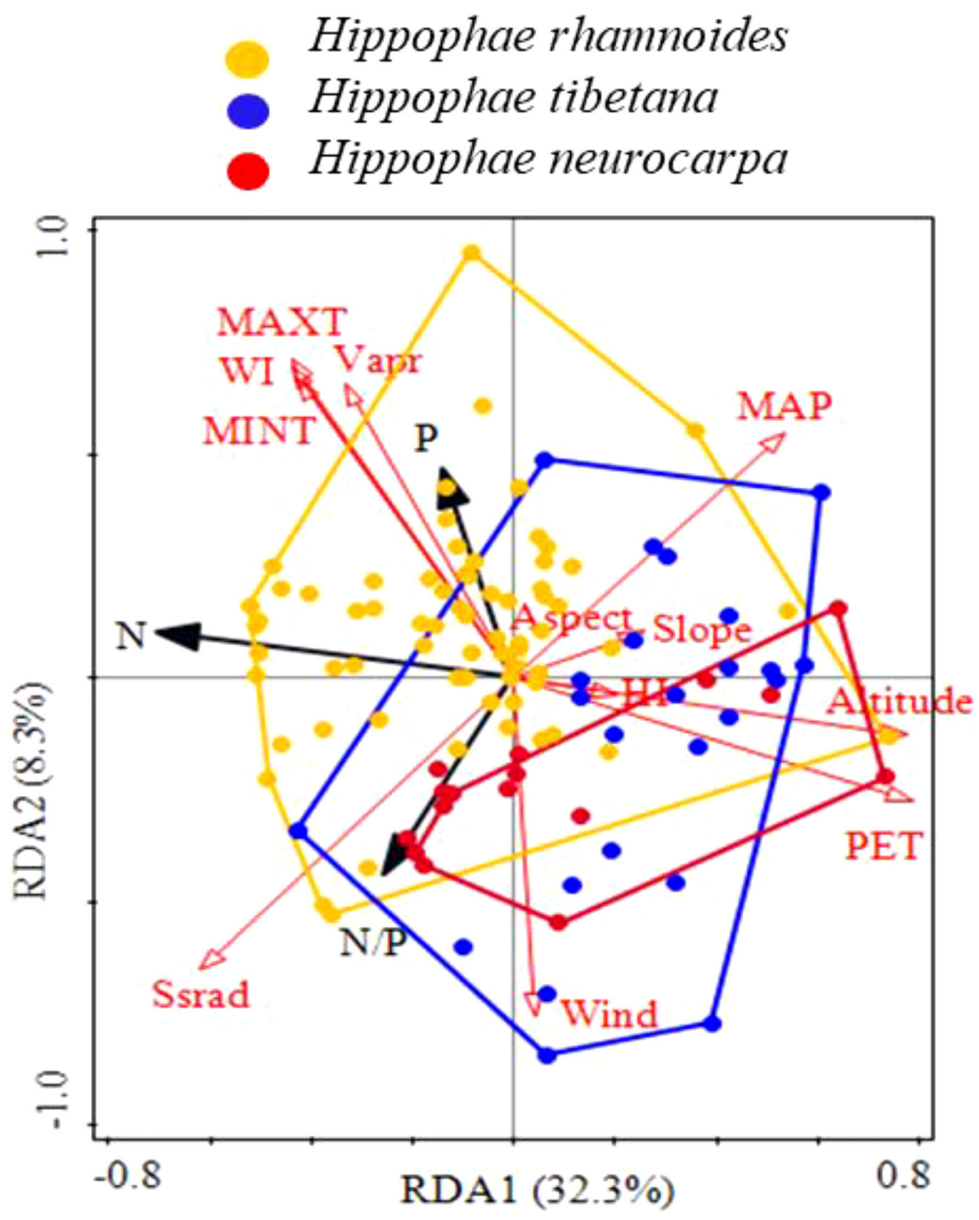
Geographical substitution distribution of leaf stoichiometric characteristics for three sea-buckthorn species. Environmental factors are indicated in red arrows, and species in black arrows. The orange polygon is for Chinese sea-buckthorn, the blue polygon for Tibetan sea-buckthorn, and the red polygon for Coastal sea-buckthorn.

The redundancy analysis confirmed that environmental factors significantly influenced the stoichiometric characteristics of sea-buckthorn leaves, for which 40.6% of their total variance was explained by the two first axes (**Figure 7**). Evidently, N was negatively correlated with potential evapotranspiration and elevation, while P was positively correlated with minimum temperature, maximum temperature, water vapor pressure, and the warmth index. Importantly, the environmental distribution of the three species (polygons in **Figure 7**) shifted towards the lower right; this being opposite to the direction of temperature increase, and also opposite to the direction of their leaf N and P concentrations increase. Hence, these results indicated that, as the temperature declined, a species shift occurred on the landscape, from Chinese sea-buckthorn to Tibetan sea-buckthorn. Moreover, there was some overlap detected in the leaf stoichiometric distribution of Coastal sea-buckthorn and Tibet sea-buckthorn, albeit the latter’s distribution was more extensive. To sum up, sea-buckthorn species with lower leaf N and P concentrations seemed more adaptable to a low-temperature environment, leading to the observed landscape pattern of plant species substitution.

## 4 Discussion

### 4.1 Adaptation strategies of leaf stoichiometric characteristics of sea-buckthorns

All sea-buckthorn species are capable of nitrogen fixation (Zhang et al., 2024), which means these plants can fix N in the atmosphere via rhizobium symbiosis to access extra N sources (Zhu et al., 2023; Xiao et al., 2024). Using our study’s results, we find that the leaf N concentration of sea-buckthorn leaves exceeds that of shrubs, and other non-legumes in China, and terrestrial plants in its northwest alpine region, in addition to plants globally (**Table 2**). Similarly, the leaf P concentration of sea-buckthorn surpassed that of non-legumes, and terrestrial leaves in China, being slightly higher than for plants globally, though it was not nearly as high as in China’s northwest alpine shrub leaves (**Table 2**). The higher N concentration of sea-buckthorns could be due to the life history of sea-buckthorn, namely the N-fixing ability of these plants, endowing them with a richer source of N for their growth and development. Plant habitat and life form will also affect the stoichiometric characteristics of leaves (Lu et al., 2023). Some studies have found that, in order to adapt to a short growing season and cold environment, plant species in alpine regions tend to have short leaf life-span and high growth rate, and are able to store more lipid P in their leaves to resist cold (Han et al., 2011; van Ommen Kloeke et al., 2012). In addition, the leaf P concentration of Chinese plants has been reported to steadily increase when moving from a humid to arid climate (Li et al., 2014). Consistent with that generalization, the P concentration of sea-buckthorn genusleaves in this study is lower than those of alpine shrubs in northwest China. The N/P ratio threshold of plant growth limited by was 14 and 16, respectively. In other words, at an N/P ratio is less than 14, plant growth at community level should be restricted chiefly by N; conversely, at an N/P ratio exceeds 16, plant growth is instead limited by P. However, when the N/P ratio is between 14 and 16, both N and P is limiting to plants (Sardans et al., 2021). According to our results, sea-buckthorn plants are therefore constrained by P limited. Consistent with this inference, other research has found that N-fixing plants are susceptible to P limitation in different ecosystems (Toro et al., 2023). This is in alignment with the findings reported in the study.

**Table 2:** Comparison between this study’s findings and other reported data.

Species	Study area	N(mg.g^-1^)	P(mg.g^-1^)	N/P	Source
This study	China	32.19	2.03	16.68	
Alpine region of northwest	Northwest China	19.77	2.45	8	He et al. (2023)
Non-legumes	299 natural temperate broadleaved deciduous shrubland	18.3 ± 1.3	1.29 ± 1.4	14.1 ± 1.4	Guo et al. (2017)
Chinese terrestrial leaves	China	18.6	1.21	14	Han et al. (2005)
Global	Worldwide	20.6 ± 12.2	1.99 ± 1.49	12.7 ± 6.82	Elser et al. (2000)

Leaf stoichiometry reflects nutrient limitations in ecosystems and plant adaptations to climate change, and geographical changes of plant nutrients are undoubtedly related to climate (Freschet et al., 2018). In the present study, the N concentration increases as the longitude, and latitude increase, while the P concentration increases as longitude increase, yet both N and P concentration s decrease with rising altitude and N/P ratio increases with latitude (**Figure 3A–E**). These findings provide compelling evidence for spatial heterogeneity in the nutrient allocation strategies of sea-buckthorn species, reflecting their unique adaptation to differing environmental conditions. Sun et al. (2017) found that *Tamarix Lour* P concentration decreased with increasing latitude. In a later study, however, Hu et al. (2017) reported that N concentration in reed leaves decreased with increasing latitude; Wu et al. (2012) found that N and N/P in *Quercus* leaves decreased with increasing latitude, while its leaf P increased with increasing latitude. These discrepancies between those studies may reflect differences between plant genera sampled and complex interactions of environmental factors. It is a well-known fact that latitudinal zonation of vegetation is mainly controlled by temperature, which has a monumental influence on plant growth and metabolic processes (Hu et al., 2017). Higher temperatures are generally beneficial to plant growth, promoting nutrient absorption and metabolic activity, which helps to augment the elemental concentration in body of plants (Heckathorn et al., 2020). Further, the ability of sea-buckthorn plants to fix N is affected by temperature. Since the nitrogen source provided by surface rhizobia is affected by temperature, when this rises it may further enhance their ability to obtain N for use by plants. This could explain why, with an increasing latitude and temperature, the leaf concentration of N and P also correspondingly increases.

### 4.2 Leaf stoichiometric spatial distribution pattern and influencing factors of *sea-buckthorn*

Our study revealed that the key factors influencing the leaf N concentration in sea-buckthorn were potential evapotranspiration and altitude, while the slope and humidity index were the primary factors affecting P concentration. Additionally, N/P ratios were predominantly influenced by annual mean solar radiation and mean annual precipitation (**Figure 6**). This is likely due to the fact that sea-buckthorn plants are predominantly distributed in regions with an average annual rainfall ranging from 400–700 mm. Potential evapotranspiration, as a critical component of the water cycle, interacts with precipitation to determine regional aridity or humidity. The synchronization of precipitation and thermal conditions in these regions creates a more favorable environment for plant growth, thereby shaping dominant tree species and influencing leaf stoichiometric characteristics (Milly and Dunne, 2016). Altitude acts as a key variable affecting vegetation growth by modulating temperature, precipitation, and humidity, which in turn influence the stoichiometry of plant leaves (Wang et al., 2021). For instance, Zhang et al. (2021) demonstrated that plant N concentration exhibited a decreasing trend along the altitudinal gradient. Zhao et al. (2014) reported a significant reduction in the overall N concentration of leaves as altitude increased. Additionally, several studies have indicated that low temperatures in high-altitude regions impose constraints on plant growth. With increasing latitude, both temperature and humidity tend to decline gradually. Certain plant species with limited tolerance may either retreat from the community due to unsuitable habitat conditions or be excluded as a result of their insufficient competitiveness, thereby influencing the levels of N and P within the ecosystem (Bergholz et al., 2017; Heckathorn et al., 2020). The slope of influences the structure and dynamics of vegetation (Sanders and Rahbek, 2012). Previous studies have demonstrated that slope and altitude affect species composition by altering habitat temperature, and humidity, as well as the spatial redistribution of solar radiation and precipitation. Additionally, leaf stoichiometric characteristics vary among different species. Slope and elevation also play a critical role in shaping the variations in N and P concentrations within plants (Cheng et al., 2023). There is a certain relationship between the humidity index and precipitation, which is crucial for plant growth. Adequate precipitation generally increases the of N and P concentrations, thereby enhancing plant growth and nutrient absorption. Moreover, high humidity promotes plant transpiration, which in turn affects leaf stoichiometry.

Collectively, water factor, topographic factor, and energy factor significantly influence the stoichiometric characteristics of sea-buckthorn leaves. Acting as the central driving force for ecosystem function, biogeochemical cycles, and life activities, water plays a pivotal role in regulating N and P element concentrations in sea-buckthorn leaves by governing energy distribution, material transport, and biological adaptation mechanisms. These findings suggest that sea-buckthorn plants adjust their nutrient allocation strategies to adapt to environmental changes.

### 4.3 Responses of leaf stoichiometric characteristics of sea-buckthorn to niche-driven species distribution

In the present study, the species N and P concentrations were ranked as follows: Chinese sea-buckthorn > Coastal sea-buckthorn> Tibetan sea-buckthorn, but reversed for their N/P ratios (16.16, 17.51, and 17.85, respectively; **Table 1**). According to the vegetation survey, the geographical distribution of sea-buckthorn encompasses temperate grassland area and the alpine meadow area located in the Loess Plateau and the eastern margin of the Qinghai-Tibet Plateau (**Figure 1**). Some studies have found that the N concentration and N/P ratio in leaves of the Loess Plateau are significantly higher than those of Chinese flora and global flora (Zheng and Shangguan, 2007). In this study, Chinese sea-buckthorn is located in the eastern margin of the Loess Plateau and the Qinghai-Tibet Plateau, so it has higher nitrogen concentration than either Tibetan sea-buckthorn or Coastal sea-buckthorn. Since all species of sea-buckthorn are N-fixers, and low temperature will affect the growth and nutrient absorption of plants, a decline N-fixation function is expected, so that the leaf N concentration of Tibetan sea-buckthorn is lower than that of Chinese sea-buckthorn and Coastal sea-buckthorn. Wang et al. (2008) investigated soil P reservoirs in China, finding more available P in the Loess Plateau than the Qinghai-Tibet Plateau, while the latter’s available P was lower than that in other regions. It could well be that where the habitats of conspecific plants differ, their plant relative demands for N and P in these local environments diverges, and their energy supply is also different; altogether, this could ultimately lead to a shift in the ecological niche (Pastore et al., 2021). It has been reported that as plant growth rates increase along with higher concentrations of N and P concentrations, the N/P ratio tends to decrease (Delgado-Baquerizo et al., 2016). Chinese sea-buckthorn and rib sea buckthorn, which are found in the transition zone between the Loess Plateau and the eastern margin of the Tibetan Plateau, exhibit higher concentrations of N and P compared to Tibetan sea-buckthorn. Consequently, the levels of N and P are relatively lower in Tibetan sea-buckthorn, which is native to the Qinghai-Tibet Plateau, resulting in a higher N/P ratio compared to the other two species of sea-buckthorn.

This study also found that the N and P concentrations of the three sea-buckthorn species showed an allometric growth pattern, and there was no significant difference in N, P distribution between Chinese sea-buckthorn and Coastal sea-buckthorn, while there was a significant difference in N, and P distribution between Tibetan sea-buckthorn (**Figure 5**). This finding aligns with the research conducted by Wang et al. (2015) on plants in the arid saline-alkali environment of Northwest China. They discovered a robust allometric relationship between N and P in leaves. However, the allometric index of Tibetan sea-buckthorn in this study differed slightly from the 3/4 index reported by Reich et al. (2010). This suggests that, relative to the P concentration in Tibetan sea-buckthorn leaves, a greater allocation of P concentration occurred. This finding implies that the growth conditions of the plants have undergone significant changes due to the increased nutrient concentration. The reason for this phenomenon may be attributed to the fact that as the temperature decreases, plants require a higher P concentration to sustain growth. Additionally, an increased P concentration can enhance the plant’s cold resistance, thereby enabling it to adapt more effectively to the changing environment. Our study revealed that the allometric exponent of Chinese sea-buckthorn, Coastal sea-buckthorn, and Tibetan sea-buckthorn exhibited a sequential increase. This finding aligns with prior studies, indicating that under more favorable climate conditions, a higher P concentration of accelerates the conversion and metabolic growth rate of plant matter, thereby resulting in a lower allometric exponent (Guo et al., 2020).

Some studies have also demonstrated that environmental factors directly influence the N and P concentrations of leaves, thereby driving intraspecific changes. Species characterized by high growth rates and strong competitive abilities tend to dominate ecosystems. Owing to variations in ecological strategies, different species exhibit distinct elemental compositions and stoichiometric characteristics, which contribute to the geographical patterns of leaf nutrient distribution via environmental filtering. This process ultimately establishes unique ecological niches for each species (Tian et al., 2024). Redundancy analysis (**Figure 7**) revealed that with the variation in temperature, the geographical shift of species distribution from the Loess Plateau to the Tibetan Plateau, Tibetan sea-buckthorn exhibited reduced competitiveness compared to the other two sea-buckthorn species in a high-nitrogen environment. Consequently, Tibetan sea-buckthorn serves as a transitional zone between Chinese sea-buckthorn and Coastal sea-buckthorn. The gradual replacement of Chinese sea-buckthorn, which has a higher P concentration in its leaves, by Coastal sea-buckthorn and Tibetan sea-buckthorn.

This substitution reflects the plant’s adaptation to its environment, both of which exhibit lower leaf P concentrations, demonstrates plant adaptation to environmental conditions. This phenomenon aligns with the regarding species composition and *biogeochemical niche hypothesis*. These changes in plant stoichiometric characteristics ultimately resulted in the establishment of a relatively stable survival strategy for various sea-buckthorn species across different environmental gradients. This, to a certain extent, reflected the differentiation of sea-buckthorn species as a group, enabling their adaptation and response to environmental conditions, and eventually leading to a niche-driven species distribution pattern. These findings for sea-buckthorn echoes those of Sardans et al. (2021) emphasizing the fundamental role of plant stoichiometric characteristics in determining both species distribution and ecosystem function.

## 5 Conclusion

In this study, we empirically analyze the spatial pattern of leaf stoichiometry, the influencing factors, and the species substitution distribution driven by the leaf stoichiometry of sea-buckthorn. The results of this study indicate that sea-buckthorn growth is constrained by P limitation, and the N concentration exhibits a significant increase with increasing latitude. There was an allometric relationship between the distribution of N and P concentrations in sea-buckthorn, with a significant allometric relationship observed in the distribution of N and P concentrations in Tibetan sea-buckthorn. Potential evapotranspiration, slope, and solar radiation were identified as the most influential factors affecting the stoichiometry of sea-buckthorn leaves, thereby facilitating niche differentiation among specific species. This analysis positioned Tibetan sea-buckthorn as a transitional zone between Chinese sea-buckthorn and Coastal sea-buckthorn, ultimately resulting in variations in leaf stoichiometry among the three sea-buckthorn species and establishing an alternative niche-driven species distribution pattern.

## Data Availability

The original contributions presented in the study are included in the article/supplementary material. Further inquiries can be directed to the corresponding author.
